# Process evaluation of the RaDIANT community study: a dialysis facility-level intervention to increase referral for kidney transplantation

**DOI:** 10.1186/s12882-017-0807-z

**Published:** 2018-01-15

**Authors:** Reem E. Hamoda, Jennifer C. Gander, Laura J. McPherson, Kimberly J. Arriola, Loren Cobb, Stephen O. Pastan, Laura Plantinga, Teri Browne, Erica Hartmann, Laura Mulloy, Carlos Zayas, Jenna Krisher, Rachel E. Patzer

**Affiliations:** 1 Department of Surgery, Division of Transplantation, Emory University School of Medicine, 1629 Pierce Dr. NE, 30322 Atlanta, Georgia USA; 2 Department of Epidemiology, Rollins School of Public Health, Emory University, Atlanta, Georgia USA; 3 Department of Behavioral Sciences and Health Education, Rollins School of Public Health, Emory University, Atlanta, Georgia USA; 4 Department of Biology, Spelman College, Atlanta, Georgia USA; 5 Department of Medicine, Division of Renal Medicine, Emory University School of Medicine, Atlanta, Georgia USA; 6 College of Social Work, University of South Carolina, Columbia, South Carolina USA; 7 Piedmont Transplant Institute, Atlanta, Georgia USA; 8 Division of Nephrology, Hypertension, and Transplant, Augusta University, Augusta, Georgia USA; 9 Southeastern Kidney Transplant Coalition, End Stage Renal Disease Network 6, Raleigh, North Carolina USA

**Keywords:** Kidney transplantation, Dialysis facility, Randomized trial, Education, Staff, Community-based participatory research, Process evaluation

## Abstract

**Background:**

The Reducing Disparities in Access to kidNey Transplantation Community Study (RaDIANT) was an End-Stage Renal Disease (ESRD) Network 6-developed, dialysis facility-level randomized trial testing the effectiveness of a 1-year multicomponent education and quality improvement intervention in increasing referral for kidney transplant evaluation among selected Georgia dialysis facilities.

**Methods:**

To assess implementation of the RaDIANT intervention, we conducted a process evaluation at the conclusion of the intervention period (January–December 2014). We administered a 20-item survey to the staff involved with transplant education in 67 dialysis facilities randomized to participate in intervention activities. Survey items assessed facility participation in the intervention (fidelity and reach), helpfulness and willingness to continue intervention activities (sustainability), suggestions for improving intervention components (sustainability), and factors that may have influenced participation and study outcomes (context). We defined high fidelity to the intervention as completing 11 or more activities, and high participation in an activity as having at least 75% participation across intervention facilities.

**Results:**

Staff from 65 of the 67 dialysis facilities completed the questionnaire, and more than half (50.8%) reported high adherence (fidelity) to RaDIANT intervention requirements. Nearly two-thirds (63.1%) of facilities reported that RaDIANT intervention activities were helpful or very helpful, with 90.8% of facilities willing to continue at least one intervention component beyond the study period. Intervention components with high participation emphasized staff and patient-level education, including in-service staff orientations, patient and family education programs, and patient educational materials. Suggested improvements for intervention activities emphasized addressing financial barriers to transplantation, with financial education materials perceived as most helpful among RaDIANT educational materials. Variation in facility-level fidelity of the RADIANT intervention did not significantly influence the mean difference in proportion of patients referred pre- (2013) and post-intervention (2014).

**Conclusions:**

We found high fidelity to the RaDIANT multicomponent intervention at the majority of intervention facilities, with sustainability of select intervention components at intervention facilities and feasibility for dissemination across ESRD Networks. Future modification of the intervention should emphasize financial education regarding kidney transplantation and amend intervention components that facilities perceive as time-intensive or non-sustainable.

**Trial registration:**

Clinicaltrials.gov number NCT02092727. Registered 13 Mar 2014 (retrospectively registered).

**Electronic supplementary material:**

The online version of this article (10.1186/s12882-017-0807-z) contains supplementary material, which is available to authorized users.

## Background

Multicomponent quality improvement interventions targeting dialysis facilities have shown demonstrated improvements in end stage renal disease (ESRD) patient outcomes and quality of care [1–4]. However, these studies tend to emphasize effect size in determining the success of complex interventions, with little regard as to why the interventions were successful or the reproducibility of the interventions in diverse contexts [5, 6]. Recent research has increasingly valued process evaluation, which focuses on the degree of implementation of an intervention, as a critical component of randomized controlled trials, especially in multisite trials where implementation may vary by setting [5, 7–9]. These evaluations are largely underreported in public health research [9, 10], but can provide important information regarding the quality of a complex intervention and its feasibility in practice [9, 11].

A widely utilized process evaluation framework, posited by Stecker and Linnan (2002) and modified by Saunders (2005), emphasizes fidelity, reach, sustainability, and context as critical dimensions of evaluating implementation of an intervention [10–12]. *Fidelity*, defined as the extent to which an intervention is delivered as intended [10], reflects the quality and integrity of the intervention and directly impacts intervention outcomes [11]. Prior studies have demonstrated intervention fidelity as a modifier in the relationship between interventions and their outcomes, with low implementation fidelity diminishing otherwise strong effect sizes [13]. Evaluating the long-term viability, or *sustainability*, of a health promotion program is critical for promoting its dissemination, adoption into practice, and maintenance in health institutions [8]. Measuring intervention sustainability also informs modification efforts by identifying which intervention components promote or hinder integration into organizational practice [8]. In order to ensure long-term beneficial outcomes of a complex intervention, it is necessary to critically assess intervention sustainability and, if needed, modify the intervention to promote sustainability [14]. The *reach* of an intervention is defined as the proportion of the intended target audience that receives each component of an intervention [10]. This measurement reflects participation rates in intervention components as well as characteristics of the participants [6]. The *context* dimension of a process evaluation refers to the evaluation of environmental, situational, or sociopolitical factors that may affect either implementation of an intervention or its intended outcomes [10]. Identifying barriers and facilitators to implementation and intervention outcomes can inform modification of complex interventions, ensuring intervention components can reliably produce desired short- and long-term outcomes in diverse contexts [5].

Results of an outcome evaluation demonstrated the effectiveness of the Reducing Disparities in Access to kidNey Transplantation Community Study (RaDIANT) multicomponent intervention in improving patient outcomes by increasing the proportion of ESRD patients referred for kidney transplant evaluation and reducing racial disparities in kidney transplant referral in the U.S state of Georgia [15]. However, a process evaluation of the RaDIANT intervention is essential for assessing the sustainability of the complex intervention, guiding modification of intervention components for dissemination of the intervention across other ESRD Networks, and strengthening our understanding of the relationships between intervention components and intended study outcomes. This paper reports the results of a process evaluation, measuring the fidelity, sustainability, reach, and context of the RaDIANT Community multicomponent quality improvement intervention aimed to increase kidney transplant referral in Georgia.

## Methods

### Intervention and process evaluation development

The RaDIANT Community Study (Clinicaltrials.gov number NCT02092727) was a randomized pragmatic trial testing the effectiveness of a multicomponent, 1-year quality improvement intervention in increasing referral for kidney transplant evaluation and reducing racial disparities in referrals from dialysis facilities in Georgia, the state with the lowest transplant rates in the nation [16, 17]. ESRD Network 6 and the community-based, multidisciplinary organization, the Southeastern Kidney Transplant (SEKTx) Coalition, led the development of the RaDIANT Community multicomponent intervention [15, 16]. Development of the RaDIANT intervention was guided by the Social Ecological Model, which considers individual, organizational, and community-level factors when planning health education interventions [18].

The multicomponent intervention was designed to be synergistic, target multiple levels (including dialysis facility leadership, staff, and patients in Georgia dialysis facilities), and emphasize transplant education for each targeted level [18]. Intervention activities were stratified as “required” or “optional” during the SEKTx Coalition’s root cause analysis conducted in 2011. Determined feasibility of implementation and their potential to address multiple barriers in access to kidney transplantation were used as designating factors. Intervention development activities and the resulting RaDIANT Community study protocol are described elsewhere in detail; the resulting intervention activities are summarized in Table 1 [15, 16, 19].

**Table 1:** Description of RaDIANT Community intervention activities^a^

Required Intervention Activities (*n* = 9)
Attending facility in-service orientation to quality improvement transplant project
Establishing dialysis facility quality improvement plan
Formation of patient and family advisory group for monthly meetings
Establishing peer mentoring program
Educational webinars for dialysis facility leadership and staff
Standard quality improvement activities and monthly monitoring of transplant referral and evaluation data
Establishing patient and family education programs
Planning a facility-wide movie night: *Living ACTS* [46]
Completing 5-Diamond Patient Safety module on transplantation [12]
Optional Intervention Activities (*n* = 5)
Kidney transplantation bulletin board
Distribution of “A Patient’s Guide to Kidney Transplant” [24]
Conduct a Transplant Education Month
Attend a “Explore Transplant” Symposium [47]
Distribution of Kidney Transplant Toolkit

^a^Facilities were required to complete 9 required intervention components and 2 out of 5 optional intervention activities

During the intervention development period, members of the SEKTx Coalition collaborated with ESRD Network 6 to design a 20- item online questionnaire (Additional File 1) based on the Steckler and Linnan process evaluation framework [10, 11], measuring the fidelity, sustainability, reach, and context of the RaDIANT Community intervention activities [16]. Questionnaire development was also guided based on prior surveys used by ESRD Network 6 to evaluate dialysis facility preferences [20].

### Data collection

At the conclusion of the one-year intervention period (in December 2014), Coalition members (with the assistance of ESRD Network 6) distributed the electronic questionnaire via HIPAA-compliant software, SurveyMonkey®, to the medical directors of 67 dialysis facilities randomized to participate in intervention activities. Dialysis facility medical directors were tasked with distributing the survey to the staff member most responsible for implementation of facility- and patient-level RaDIANT intervention activities at their dialysis facility. Staff were asked to report facility-level participation in each intervention activity (fidelity), the helpfulness (5-point Likert Scale) of each activity (sustainability), whether or not they were willing to continue each activity after the 12 month intervention period (sustainability), which educational resources were administered by their facility to patients (context), and which transplant process tracking activities were used by the dialysis facility (context). Respondents were also asked to identify potential barriers preventing patients from beginning or completing the transplant evaluation process (context), as well as provide suggestions for improving specific components of the intervention (sustainability). All staff members who completed the survey reported their staff title and 6-digit facility provider number. Survey data were linked via unique provider number to facility-level data on patients seen at dialysis facilities (reach) and patient referrals made from a dialysis facility to a Georgia transplant center during the intervention year (2014). Facility-level referral data were collected as previously described [15, 16, 21].

### Inclusion/exclusion criteria

Dialysis facilities selected for participation in the RaDIANT Community Study had either low referral for transplantation or racial disparities in transplant referral, as previously described [15]. To reduce variability in self-reported implementation of intervention components, we restricted our survey population to include one unique response from each of the 67 intervention facilities. Selection methods were informed by previous studies assessing transplant education practices at dialysis facilities [22]. If duplicate responses were received from the same staff member at a facility, a unique response was selected based on survey completion (completion of at least 50% of the survey). If multiple staff members completed a survey from a facility, we selected the unique facility response based on 1) survey completion and 2) facility role of the staff member completing the survey. Clinic managers, social workers, and dialysis nurses, as compared to facility administrators and administrative assistants, were prioritized as having more responsibility in implementing the RaDIANT intervention, as they have been identified previously as staff members most responsible for transplant education at dialysis facilities [22, 23].

### Data analysis

Table 2 describes the measures used for process evaluation of the RaDIANT Community intervention. Counts and frequencies were tabulated for all categorical variables, and means and standard deviations were calculated for all continuous variables. High participation in a RaDIANT intervention component was defined *a priori* as having at least 75% participation in the activity. A fidelity index was calculated by summing the number of staff-reported required and optional intervention components by a facility, ranging 0 to 14. As full implementation of the RaDIANT intervention required completion of 11 total intervention components (9 required components and 2 optional components), we defined high fidelity to the RaDIANT intervention a priori as having a fidelity index of 11 or greater, with low fidelity defined as having a fidelity index of 10 or less. Simple linear regression was used to determine the association between fidelity index (range 0–14) and mean difference in proportion of patients referred at baseline (2013) and in the intervention year (2014). All results were considered statistically significant at the *P* < 0.05 level. SAS 9.4® (Cary, NC) statistical software was used for all analyses.

**Table 2:** Process evaluation measures for the RaDIANT Community intervention

Dimension	Process Evaluation Questions	Measurement
*Fidelity*	To what extent was the intervention delivered as planned?	Facility-level participation (Y/N) in each required and optional intervention component and in all required and optional intervention activities^a^ Composite fidelity index of the number of intervention components completed (0–14)
*Sustainability*	To what extent are intervention activities perceived as helpful? To what extent are dialysis facilities willing to continue intervention activities indefinitely? How can the intervention be improved for dissemination?	Staff-perceived helpfulness of intervention activities (Likert scale) Willingness to continue intervention activities (yes/no) based on perceived helpfulness Open-ended questions regarding suggestions for improving select intervention activities
*Reach*	To what extent did its intended audience receive the intervention?	Proportion of patients receiving each patient-level intervention component and the full multicomponent intervention^b^
*Context*	What barriers may prevent patients at intervention facilities from beginning or completing the transplant evaluation process? What non-intervention educational materials or resources were provided to patients at intervention facilities?	Itemized checklist at end of questionnaire Facility-level distribution (Y/N) of non-RaDIANT educational resources to dialysis patients
*Linking process data to intervention outcomes*	To what extent does facility level referral for kidney transplantation vary by implementation fidelity among intervention facilities pre- and post-intervention?	Association between fidelity index (0–14) and mean difference in proportion of patients referred for kidney transplantation in the 2014 intervention year

^a^ Intervention facilities were required to complete 9 mandatory intervention activities, as well as 2 “optional” intervention activities

^b^ Receipt of an intervention component is defined as receiving care at a facility that adopted the intervention component

## Results

### Study population

97% of the 67 dialysis facilities randomized to participate in intervention activities during the intervention period had at least 1 staff member complete the questionnaire, with 94 staff members in total completing the survey. 35% (*N* = 23) of facilities had multiple responses to the questionnaire (range: 1 to 4 responses per facility). Out of 29 responses excluded from our analysis, 86% (*n* = 25) were duplicate responses from the same staff member at a facility. The remaining 14% (*n* = 4) of responses were excluded from facilities where two independent staff members completed the survey for a facility (no more than 2 unique staff members completed the survey per facility). More specifically, 2 staff responses were excluded due to having completed less than 50% of the survey, 1 response was from a facility administrator (compared to a social worker), and 1 response was from a social worker (compared to a clinic manager). There was no significant differences in mean fidelity index between responses that were included for analyses (*n* = 2, mean (SD): 12.5 (2.1)) and complete responses that were excluded (*n* = 2, mean (SD): 5.6 (6.8)), among the 2 facilities with multiple complete responses. Among the staff members that were included in our analyses (*n* = 65), 50.8% were social workers, 15.4% were clinic or nurse managers, 10.8% were facility administrators, 4.6% were administrative assistants, and 4.6% were nurses. Table 3 describes additional facility and patient-level characteristics of intervention facilities represented in the survey population.

**Table 3:** Staff, patient, and facility-level characteristics of survey population

Characteristics	*n* = 65
Survey Population Characteristics	
Staff role^a^	
% Social Worker	50.8
% Clinic (nurse) manager	15.4
% Facility administrator	10.8
% Administrative assistant	4.6
% Nurse	4.6
Facility Characteristics^b^	
No. of patients per facility, mean ± SD	44.6 ± 24.3
No. of staff, mean ± SD	9.4 ± 5.6
% For profit	88.1
% crude referral in lowest 50th percentile	64.6
% within-facility racial disparity in kidney transplant referrals at baseline (2013)	35.4
Characteristics of patients within facilities^b^	
Age (years), mean ± SD	60.2 ± 6.2
% White	32.8
% Black	65.8
% Hispanic	2.0
% Hemodialysis modality	94.8
% Uninsured at ESRD start	10.1
% Medicaid only at ESRD start	12.9
% Unemployed	69.2
Average time on dialysis (years), mean ± SD	5.0 ± 1.2
% Receiving no pre-ESRD nephrology care	25.8
% Not informed of transplant options	3.2
% With diabetes	54.4
% With hypertension	92.2
% With arteriovenous fistula	29.9
Average count of comorbidities, mean ± SD	2.8 ± 0.8

^a^13.8% of respondents did not report a staff role (*n* = 9)

^b^2008–2011 baseline characteristics of selected Georgia dialysis facilities randomized to participate in RaDIANT intervention

### Fidelity

Of the 65 facilities that were represented in the survey population, 50.8% (*n* = 33) reported to have adhered fully to the RaDIANT Community intervention requirements, with 45.0% (*n* = 29) of facilities that adhered fully to the RaDIANT multicomponent intervention participating in all intervention activities (including all 5 optional activities) (Table 1). Furthermore, 4.6% (*n* = 3) of facilities randomized to the RaDIANT intervention reported to not have participated in any intervention activity; 2 non-participating facilities saw a decrease in the proportion of patients referred between baseline and the intervention period, and 1 facility experienced a minimal increase in referral that was below the mean difference in referral among all intervention facilities [15]. Among the facilities that adhered to the multicomponent intervention (*n* = 62), 97.0% completed more activities than was required (more than two “optional” activities). The median and mean fidelity index was 12 (IQR = 3) and 11.1 (SD = 3.4), respectively. On average, facilities completed 7.6 (SD = 2.3) of 9 required intervention activities, and 3.6 (SD = 1.3) of 5 optional intervention activities.

Across the 65 intervention facilities, 8 out of 9 required activities and 3 out of 5 optional activities exhibited high participation (greater than 75% participation). The most often implemented required intervention activities included organizing an in-service staff orientation (92.3%), participation in standard quality improvement and monthly monitoring activities (92.3%), and participation in staff educational webinars (92.2%). The top three optional intervention activities implemented were distribution of “A Patient’s Guide to Kidney Transplant” [24] (84.6%), creation of a transplant education bulletin board or poster (75.4%), and creation of a comprehensive kidney transplant toolkit (75.4%). The least implemented required intervention activity was formulation of a peer-mentoring program (67.7%), and the least implemented “optional” intervention activity was hosting of a facility-wide transplant education month (56.9%). Table 4 summarizes staff-reported participation in RaDIANT intervention activities.

**Table 4:** Staff-reported facility participation in RaDIANT intervention components^a^

Required Intervention Activities (*n *= 9)	Staff- reported facility participation at study end (*n* = 65 facilities), *n* (%)
In-service staff orientation	60 (92.3)
Standard QI activities	60 (92.3)
Educational webinars	60 (92.3)
Quality improvement plan	58 (89.2)
5 Diamond module	57 (87.7)
Patient and family education programs	54 (83.1)
Movie Night: *Living ACTS*	52 (80.0)
Patient and family advisory group	46 (70.8)
Peer mentoring program	44 (67.7)
Optional Intervention Activities (*n* = 5)	
A Patient’s Guide to Kidney Transplant	55 (84.6)
Transplant bulletin board/poster	49 (75.4)
Kidney Transplant toolkit	49 (75.4)
Transplant symposium	42 (64.6)
Transplant Education Month	37 (56.9)
Fidelity Index^b^, mean ± SD	11.1 ± 3.4

^a^Facilities were required to complete 9 required intervention components and 2 out of 5 optional intervention activities

^b^Composite index of the number of intervention components (required and optional) implemented by a dialysis facility (range: 0 to 14)

### Sustainability

Table 5 summarizes sustainability results. Nearly two thirds (63.1%) of facilities reported the majority (5 or greater) of RaDIANT intervention activities being helpful or very helpful, with 22.0% of these facilities reporting all RaDIANT required intervention activities as helpful or very helpful. Among the facilities that implemented every activity, the top activities perceived as helpful or very helpful, included the in-service staff orientation (86.4%), staff educational webinars (79.7%), and patient and family education programs (75.5%). Activities perceived as most unhelpful or ineffective include the *Living ACTS* movie night (35.3%) [25, 26], standard quality improvement and tracking activities (45.8%), and the formulation of a patient and family advisory group (51.1%).

**Table 5:** Sustainability of the RaDIANT Community intervention

Intervention activity^a^	Perceived helpfulness of intervention activity^b^	Willingness to continuing intervention activity^b,c^
	%	%
In-service staff orientation	85.0	54.9
Educational webinars	78.3	12.8
Patient and family education programs	74.1	60.0
Quality improvement plan	70.7	14.6
5 Diamond Patient Safety module	66.7	0
Peer mentoring program	65.9	69.0
Movie Night: *Living ACTS*	63.5	36.4
Standard QI activities	53.3	50.0
Patient and family advisory group	47.8	31.8

^a^Optional intervention activities were not measured for staff-perceived helpfulness

^b^Excludes missing responses (*n* = 1) and “did not participate” in the denominator

^c^Among participating intervention facilities that found activities helpful or very helpful

Although no facility was willing to continue all required intervention activities, 90.8% of facilities were willing to continue at least one activity indefinitely. Top activities that facilities were willing to continue, among facilities that implemented the activity and found the activity helpful or very helpful, included establishing a peer-mentoring program (69.0%), patient and family education programs (60.0%), and an in-service staff orientation (54.9%). Activities that were least likely to be continued include the 5 Diamond Patient Safety module [12] (0%), staff educational webinars (12.8%), and implementing a standard quality improvement plan (14.6%).

When asked about topics that should be emphasized in future educational webinars, staff frequently reported including webinars pertaining to financial barriers to kidney transplantation, such as financial education for patients and reducing transportation barriers. For example, one staff member wrote that webinars should focus on “specific financial information regarding transplantation that many patients ask and are fearful of”. Another frequent theme in staff responses was concerning compliance with the kidney transplant process. As one staff member wrote, “Promoting compliance with the transplant evaluation process could have been discussed earlier than it was. This would have been beneficial to assisting patients with any barriers/concerns for the process”.

### Reach

We estimate that 4166 patients received care at intervention facilities during the intervention year (2014); this estimate represents prevalent patient counts obtained monthly from all intervention facilities in 2014. Among these patients, 50.9% received care at facilities adhering to the full RaDIANT intervention. The top three patient-level intervention components received by dialysis patients at intervention facilities included receipt of “A Patient’s Guide to Kidney Transplant” (82.3%), promotion of patient and family educational programs (80.5%), and provision of a facility-wide movie night for *Living ACTS* (77.5%). The least received patient-level intervention components included engagement in a transplant education month (54.4%), attending an “Explore Transplant” symposium [27, 28] (64.7%), and participation in a peer-mentoring program (66.3%).

### Context

To inform potential modification of the RaDIANT intervention for improved access to later steps in the transplant process, we assessed staff-perceived barriers that may have prevented referred patients receiving intervention materials from beginning or completing the kidney transplant evaluation (Table 6). The most frequency reported barriers to starting or completing a transplant evaluation once referred were financial in nature, including patients’ socioeconomic status (73.9%), inability to afford medications after transplant (72.3%), and patient perceived fundraising requirements (60.0%). Additional barriers listed by staff include lack of transportation to a transplant center (56.9%), contentment with existing dialysis treatment (53.9%), and loss of interest in continuing the evaluation process once referred (53.9%). Table 6 summarizes the top three logistical, emotional/motivational, knowledge, health, financial, and demographic barriers reported by staff.

**Table 6:** Staff-perceived barriers to accessing kidney transplant evaluation among referred ESRD patients^a^

	Staff-reported barrier, *n* (%)
Logistical Barriers	
Lack of transportation to the transplant center	37 (56.9)
Distance to transplant center	33 (50.8)
Timing of appointment (i.e., conflicts with work or dialysis schedule)	10 (15.4)
Emotional/Motivational Barriers	
Evaluation doesn’t seem urgent as patient does not mind dialysis	35 (53.9)
Lost interest in continuing the evaluation process after initial appointment	35 (53.9)
Anxiety about appointment or transplant procedure itself	26 (40)
Knowledge Barriers	
Low health literacy	33 (50.8)
Lack of understanding about the transplant process itself	27 (41.5)
Low literacy	25 (38.5)
Health Barriers	
Cardiovascular disease	36 (55.4)
Decreased functional status/needing assistance with daily activities	29 (44.6)
Diabetes	21 (32.3)
Financial Barriers	
Cannot afford medications post-transplant	47 (72.3)
Patient perceived fundraising requirements	39 (60)
Cannot afford co-pay	30 (46.2)
Demographic Barriers	
Socioeconomic status	48 (73.9)
Age	27 (41.5)
Race	8 (12.3)

^a^Top three reported barriers in each subcategory

To determine the extent to which intervention facilities were encouraging transplant patient education outside of RaDIANT guidelines, staff were asked to report the general transplant, transplant process, and financial and social support educational resources administered to dialysis patients by their facility. Among our survey population, 92.3% of intervention facilities reported the use of at least one non-RaDIANT educational material; the mean number of non-RaDIANT educational resources administered was 4.5 (SD = 3.2) resources. Among select general transplant education resources administered to patients, the “Explore Transplant” website and associated educational materials were distributed most frequently (60.0%). The top provided education resource specifically regarding the transplant process was a local transplant center’s eligibility criteria for a kidney transplant (73.9%), and the top financial or social support educational resource provided by facilities was Georgia Transplant Foundation’s “Financial Resources” material (58.5%) [29]. When staff were asked which transplant-related educational resources were most beneficial, respondents (*n* = 24) most frequently cited educational materials that addressed financial barriers to kidney transplantation (33.3%).

### Linking process data to outcomes

We also sought to identify whether variation in implementation of the RaDIANT intervention and its components influenced study outcomes, namely the change in facility-level transplant referral, stratified by patient race, at the conclusion of the 2014 intervention year. Implementation fidelity did not significantly affect the increase in proportion of patients referred across Georgia dialysis facilities post-intervention (β ± standard error = 0.001 ± 0.005, *p* = 0.81).

## Discussion

We previously demonstrated the effectiveness of the RaDIANT Community Study’s multicomponent intervention in increasing racial equity and access to referral for kidney transplantation across Georgia dialysis facilities [15]. Results of our process evaluation indicated high implementation fidelity of the RaDIANT multicomponent intervention across participating dialysis facilities with low crude referrals or racial disparities in referral, and with a slight majority of dialysis facilities reporting full adherence to RaDIANT intervention requirements. This result is notable given the large number of facilities included in our study population, the complex nature of our intervention, and the community-based aspect of implementation [7, 30]. We also report high sustainability of the RaDIANT multicomponent intervention, with two thirds of participating dialysis facilities perceiving the majority of RaDIANT intervention activities as helpful or very helpful, and nearly all participating facilities willing to continue at least one required intervention component. Highly implemented intervention components included the in-service staff orientation and patient/family education programs, with staff perceiving these components as most sustainable for future implementation. The helpfulness and sustainability of these education-based components reflects an aim of the RaDIANT Community Study in increasing access to kidney transplantation in Georgia through patient-, provider-, and system-level transplant education [16].

Although there was considerable variability in fidelity of implementation across facilities, this variability did not significantly influence study outcomes. Particularly, facilities that did not implement the full RaDIANT intervention still achieved significant increases in facility-level referral. This finding suggests that “flexible” adaptation and implementation of the RaDIANT intervention in poor performing facilities outside of Georgia may be feasible, with facility-level contextual differences likely influencing implementation fidelity but not affecting the overall effectiveness of the intervention [31–35]. However, prior studies have shown that an increase in use of transplant educational materials is associated with an increased access to subsequent steps of the transplant process, including waitlisting for a kidney transplant [22, 27, 36]. Therefore, we encourage any dialysis facility adopting the RaDIANT intervention to implement as many of the “required” RaDIANT intervention activities as possible, utilizing our feasibility and sustainability analysis as a guide for selecting intervention components to implement in their respective centers.

Although recent studies have identified patient-perceived barriers to starting and completing an evaluation [37, 38], no prior studies have examined dialysis staff-reported patient barriers in starting or completing kidney transplant evaluation. Facilities largely emphasized financial concerns as barriers in access to evaluation in the Georgia ESRD population, with staff perceiving educational materials addressing financial barriers to transplantation as most beneficial. Furthermore, staff recommended increased use of financial educational resources as a potential improvement to the RaDIANT intervention. These findings suggest that the RaDIANT intervention may not fully address financial barriers in access to kidney transplantation, barriers that persist in the Georgia ESRD population [17, 21, 37–40]. Although socioeconomic factors, such as insurance, neighborhood poverty, and income level, are less modifiable patient-level barriers to kidney transplantation access, higher knowledge of financial resources available to ESRD patients may still serve to increase transplant access [41]. We recommend modification of the RaDIANT intervention to increase distribution of financial education with regard to kidney transplantation for both patients and providers, as this may facilitate increased access to steps in the kidney transplantation process following referral.

Intervention components that staff were most unwilling to continue appeared to be activities that either posed systematic logistical constraints (e.g. patient and family advisory groups) or activities that required a significant time commitment (e.g. staff educational webinars). Conversely, intervention components that staff were more interested in continuing appeared to require smaller time commitments or were more self-sustaining, including the peer mentoring programs and patient/family educational programs. We attribute these findings to dialysis facility staff’s busy schedules and potentially heavy workloads, with time-intensive intervention activities being less sustainable [32, 42]. Furthermore, implementing time-intensive interventions may create discordance with existing clinical practices aimed to meet Centers for Medicare and Medicaid Services (CMS) mandated quality measures for dialysis care [43, 44]. Future modification efforts should consider feasibility of adopting intervention components into practice, amending logistically challenging or time-intensive intervention components to maximize sustainability and likelihood of integration into the dialysis clinic workflow [32].

There are limitations to our process evaluation. First, the use of staff-reported process data may have resulted in social desirability bias due to over-reporting of adherence to intervention requirements. We believe this bias may have resulted in overestimation of our fidelity measures. However, utilization of self-reported data for measuring fidelity was more feasible given resources available and the community-based nature of our study [10]. Second, we restricted survey responses to one staff response per facility, and implementation of some intervention components may have differed within facilities. However, we believe that our selection methods eliminated ambiguity due to multiple responses from a given facility, as well as afforded increased validity of our results by utilizing responses from staff members most responsible for implementation of RaDIANT educational materials. Third, we were unable to obtain patient-level data regarding receipt or helpfulness of patient-level intervention components, which may have affected the validity of our reach and sustainability measures. Fourth, due to the nature of the RaDIANT intervention being multicomponent, it is not possible to parse which components were most effective [15]. However, previous studies on educational materials utilized in the RaDIANT intervention, including the patient and family education programs and the *Living ACTS* movie and booklets, have shown effectiveness in increasing knowledge and interest in both kidney transplantation and organ donation [26, 45–47].

## Conclusions

The RaDIANT multicomponent intervention was implemented with high fidelity, demonstrated potential for sustainability among Georgia dialysis facilities, and may be feasible for dissemination to facilities across ESRD Networks outside of Georgia. Future modification efforts should include adopting more intervention components aimed to address financial concerns regarding kidney transplantation, amending intervention components that are perceived by dialysis facilities as time-intensive or logistically challenging, and promoting patient- and facility-level components that staff perceive as both helpful and sustainable.

## Additional file

Additional file 1: ESRD Network 6 RaDIANT Process Evaluation Questionnaire. 20- item questionnaire designed by the Southeastern Kidney Transplant (SEKTx) Coalition to measure fidelity, reach, sustainability, and context of the RaDIANT intervention (DOCX 25 kb)

## Abbreviations

ACTS: About Choices in Transplantation and Sharing
CMS: Centers for Medicare and Medicaid Services
ESRD: End stage renal disease
RaDIANT: Reducing Disparities in Access to kidNey Transplantation
SEKTx: Southeastern Kidney Transplant
