# Supplementary material for: Process evaluation of the RaDIANT community study: a dialysis facility-level intervention to increase referral for kidney transplantation
Source: BMC Nephrol. 2018 Jan 15;19:13. doi: 10.1186/s12882-017-0807-z (PMC5769303; doi:10.1186/s12882-017-0807-z)
Supplement: Additional file 1: — ESRD Network 6 RaDIANT Process Evaluation Questionnaire. 20- item questionnaire designed by the Southeastern Kidney Transplant (SEKTx) Coalition to measure fidelity, reach, sustainability, and context of the RaDIANT intervention (DOCX 25 kb) [file 12882_2017_807_MOESM1_ESM.docx]

**ESRD Network 6 Process Evaluation for the 2014 Increasing Transplant Referral Project**

*Emailed by ESRD Network Leadership to Dialysis Facility Medical Directors*

*Email will include website link for SurveyMonkey survey*

The purpose of this survey is to measure your degree of satisfaction with the 2014 Transplant Quality Improvement Project interventions and activities. Your facility participated in this project to improve transplant coordination through increasing kidney transplant referrals and reduce racial disparity in transplant referrals. The survey should take approximately 10 minutes to complete and should be completed by the staff with the most involvement in quality improvement projects and transplant activities. We value your feedback and appreciate you taking the time to complete this survey. Thank you.

**Title of person completing form (**e.g., Medical Director): _____________________________

**Dialysis Facility Name:** ________________________________________________________

**Dialysis Facility Provider Number:** ____________________________________________

**Dialysis Facility Address: _______**____________________________________________

**Dialysis Facility Phone:** __________________ **Dialysis Facility Fax: __________________**

1. As part of the Transplant Project, did your facility form a Patient and Family Advisory Group to assist with the project goals, objectives and interventions?

Yes ___________ No __________

1. If you answered yes, in what month was the patient advisory group formed? What is the frequency of group meetings?

Month _________ Meeting Schedule (e.g., monthly) __________

1. Is the Patient and Family Advisory Group still active?

Yes ___________ No __________

1. Did your facility conduct an in-service/orientation with staff regarding the intervention materials and your commitment to increasing patient referrals for transplant?

Yes ___________ No __________

1. If you answered yes, in what month was the in-service/orientation held?

Month _________

1. In the table below, please mark which of the educational resources that are administered to patients regarding the referral process or transplantation itself. If a resource you provide is not listed, please list in the “Other” row at the bottom of the table.

| Educational Resource | Mark if Yes |
| --- | --- |
| General Transplant Education | |
| Poster or bulletin board focusing on transplantation |  |
| Downloaded and guided patients through decision aid “iChoose Kidney” |  |
| “Explore Transplant” materials and website with patient |  |
| Downloaded/distributed “A Patient’s Guide to Kidney Transplants” to patients |  |
| Distributed Kidney Funds Flyer “Treatment Option: Kidney Transplant” |  |
| Designated/conducted a Transplant Education Month |  |
| National Kidney Foundation’s “Real Stories: Real people, real kidney stories” with patient |  |
| Distributed any of the following brochures: | |
| “Living ACTS: A Giving Act” |  |
| “The Little Book of Giving ACTS: about choices in transplantation and sharing” |  |
| “Why kidney patients get transplants” |  |
| “Why people donate their kidneys” |  |
| The Transplant Process | |
| Local Transplant Center transplant eligibility criteria |  |
| Transplant medication iPad application |  |
| American Association of Kidney Patients’ “Healthy Life After Transplant” |  |
| Financial Support | |
| Georgia Transplant Foundation’s “Financial Resources" |  |
| Health Resources and Service Administration’s  “Guide to Transplantation” |  |
| United Network of Organ Sharing’s  “What Every Patient Needs to Know” |  |
| Social Support | |
| National Kidney Foundation’s: “Peer Mentoring Program” |  |
| Georgia Transplant Foundation’s “Peer Mentoring Program” |  |
| Other (please specify publishing organization and title): |  |

1. **In the table below, please mark which steps in the transplant process that your center routinely tracks for your patient population. If you do track a particular step, please specify how often (e.g., annually, monthly, etc.) and please estimate the percentage of your patient population that completed that particular step.**

| Transplant Evaluation Step | Mark if Tracked | How often? | Percentage | |
| --- | --- | --- | --- | --- |
| Patient educated about transplantation |  |  |  | |
| Patient referred for transplant evaluation |  |  |  | |
| Patient starts the evaluation process |  |  |  | |
| Patients completes the evaluation process |  |  |  | |
| Patient is placed on waitlist |  |  |  | |
| Patient is placed on active versus inactive waitlist |  |  | Active | In-active |

1. Did your facility implement a peer mentoring program to connect patients and/or families with transplant recipients or waitlisted dialysis patients? If ‘Yes’ please answer questions 9 and 10.

Yes ___________ No __________ N/A __________

1. If you answered yes, in what month was the peer mentoring program implemented and how often did the mentors visit?

Month __________

How many times did the mentor meet with the mentees? __________

N/A __________

1. Did your facility use the Georgia Transplant Foundation’s Peer Mentoring services?

Yes ___________ No __________

1. As part of the intervention, your facility was required to participate in monthly webinar sessions. Please rate your level of agreement that the following webinar topics were beneficial to the facility, staff, and patients.

|  | Extremely Not Helpful | Not Helpful | No Effect | Helpful | Very Helpful | Did  Not  Participate |
| --- | --- | --- | --- | --- | --- | --- |
| Improving Transplant Coordination-Kickoff Project (January 2014) |  |  |  |  |  |  |
| The Mentor Project/Georgia Transplant Foundation (February 2014) |  |  |  |  |  |  |
| The Explore Transplant Program (March 2014) |  |  |  |  |  |  |
| Transplant Center Coordinators (April 2014) |  |  |  |  |  |  |
| Living ACTS-Choices in Transplantation and Sharing (May 2014) |  |  |  |  |  |  |
| Elevator Pitch-Facility Presentation (June 2014) |  |  |  |  |  |  |
| Best Practices to Improve Transplant Coordination (July 2014) |  |  |  |  |  |  |
| Transplant Patient Stories (August 2014) |  |  |  |  |  |  |
| How to Promote Compliance to the Transplant Evaluation and Completion process (September 2014) |  |  |  |  |  |  |
| Innovative Strategies to Increase Transplantation in Southeastern Transplant Centers (October 2014) |  |  |  |  |  |  |
| The Navigation Study: A Pilot Study to Determine the Impact of Individualized Support to Reduce Waitlisted Time for Transplants (November 2014) |  |  |  |  |  |  |
| Wrap-Up and Sustainability (December 2014) |  |  |  |  |  |  |

1. Are there any other topics that were not part of the webinar sessions that you think should be covered in the future? Should some topics have been presented earlier/later in the intervention period?
2. Did your facility conduct a patient and family education session on transplantation?

Yes ___________ No __________ N/A __________

1. In what month was the session was conducted?

Month __________ N/A _________

1. Was a local transplant center coordinator invited to speak at the session in an effort to provide further insight about the transplant evaluation process?

Yes ___________ No __________ N/A __________

1. Patients and/or staff from your facility had the option to participate in the Georgia Transplant Foundation education conference, “Trends in Transplant.” Did anyone from your facility attend this conference? If yes, please answer question 18.

Yes ___________ No __________ N/A __________

1. If so, in talking with your facility’s participants, what topics were of interest and what transplant-related educational resources were most beneficial?
2. Despite intervention activities, some referred patients have not yet begun or completed the evaluation process. In your opinion, what are the most common barriers that prevent referred patients from beginning or completing the transplant evaluation process? Check all that apply.

| **Transplant Evaluation Barriers** | |
| --- | --- |
| ***Logistical Barriers*** | |
| Lack of transportation to the transplant center |  |
| Distance to transplant center |  |
| Lack of childcare during evaluation appointment |  |
| Lack of communication between patient and staff at transplant center regarding appointment |  |
| Timing of appointment (i.e., conflicts with work or dialysis schedule) |  |
| Confusion regarding day and time of appointment |  |
| Other |  |
| ***Emotional/Motivation Barriers*** | |
| Patient feels uncomfortable or unwelcome at transplant center |  |
| Anxiety about appointment or transplant procedure itself |  |
| Fear of receiving bad news at the appointment |  |
| Worried about not “passing” medical tests at the appointment |  |
| Fear that no one will serve as a donor |  |
| Lost interest in continuing the evaluation process after initial appointment |  |
| Evaluation doesn’t seem urgent as patient does not mind dialysis |  |
| Religious or other beliefs about donated organs? |  |
| Patient does not want to lose friends made at dialysis center |  |
| Other |  |
| ***Knowledge Barriers*** | |
| Patients believe that starting dialysis or being referred means that they are already waitlisted |  |
| Lack of understanding about the purpose of the evaluation |  |
| Lack of understanding about the transplant process itself |  |
| Lack of understanding about the transplant center’s scheduling system (i.e., impact of not showing up to an appointment) |  |
| Lack of understanding about the benefits of transplant |  |
| Low literacy |  |
| Low health literacy |  |
| Other |  |
| ***Health Condition Barriers*** | |
| Diabetes |  |
| Hypertension |  |
| Anemia |  |
| Cardiovascular disease |  |
| Decreased functional status/needing assistance with daily activities |  |
| Other (cancer) |  |
| ***Financial Barriers*** | |
| Type of insurance |  |
| Cannot afford co-pay |  |
| Cannot afford medications post-transplant |  |
| Patient perceived fundraising requirements |  |
| Other |  |
| ***Demographic Barriers*** | |
| Socioeconomic status |  |
| Age |  |
| Race |  |
| Language |  |
| Other |  |

1. Please rate your level of agreement that the following intervention activities were beneficial overall to the facility, staff, and patients. Mark ‘N/A’ for the activities in which your facility did not participate

|  | Extremely Not Helpful | Not Helpful | No Effect | Helpful | Very Helpful | Did  Not  Participate |
| --- | --- | --- | --- | --- | --- | --- |
| Dialysis facility formulated Improvement Plan |  |  |  |  |  |  |
| Patient-Family Advisory Group |  |  |  |  |  |  |
| Peer Mentoring Program |  |  |  |  |  |  |
| Monthly Webinars |  |  |  |  |  |  |
| Submission of patient-specific transplant referral forms |  |  |  |  |  |  |
| Staff orientation on transplant |  |  |  |  |  |  |
| Patient and Family Education Session |  |  |  |  |  |  |
| Movie Night: Living ACTS DVD |  |  |  |  |  |  |
| 5 Diamond Module |  |  |  |  |  |  |
| Participation in Georgia Transplant Foundation symposium |  |  |  |  |  |  |
| Distribution of ‘A Patient’s Guide to Kidney Transplant’ |  |  |  |  |  |  |
| Creation of comprehensive Kidney Transplant Toolkit |  |  |  |  |  |  |

1. **Which activities is your facility willing to continue? Mark all that apply.**

|  | YES |
| --- | --- |
| Formulation of dialysis facility Improvement Plan |  |
| Patient-Family Advisory Group |  |
| Peer Mentoring program |  |
| Monthly webinars |  |
| Submission of patient-specific transplant referral forms |  |
| Staff orientation on transplant |  |
| Patient and family education session |  |
| Movie Night: Living ACTS DVD |  |
| 5 Diamond Module |  |
| Participation in Georgia Transplant Foundation symposium |  |
| Distribution of ‘A Patient’s Guide to Kidney Transplant’ |  |
| Creation of comprehensive Kidney Transplant Toolkit |  |
| Other ongoing activities (please describe) |  |
